# Multimorbidity Patterns in Elderly Primary Health Care Patients in a South Mediterranean European Region: A Cluster Analysis

**DOI:** 10.1371/journal.pone.0141155

**Published:** 2015-11-02

**Authors:** Quintí Foguet-Boreu, Concepción Violán, Teresa Rodriguez-Blanco, Albert Roso-Llorach, Mariona Pons-Vigués, Enriqueta Pujol-Ribera, Yolima Cossio Gil, Jose M. Valderas

**Affiliations:** 1 Institut Universitari d’Investigació en Atenció Primària Jordi Gol (IDIAP Jordi Gol). Universitat Autònoma de Barcelona, Gran Via Corts Catalanes, 587 àtic, Barcelona, Spain; 2 University of Girona, Carrer Emili Grahit, 77, Girona, Catalonia, Spain; 3 Health Services & Policy Research Group, School of Medicine, University of Exeter, Exeter, United Kingdom; Sudbury Regional Hospital, CANADA

## Abstract

**Objective:**

The purpose of this study was to identify clusters of diagnoses in elderly patients with multimorbidity, attended in primary care.

**Design:**

Cross-sectional study.

**Setting:**

251 primary care centres in Catalonia, Spain.

**Participants:**

Individuals older than 64 years registered with participating practices.

**Main outcome measures:**

Multimorbidity, defined as the coexistence of 2 or more ICD-10 disease categories in the electronic health record. Using hierarchical cluster analysis, multimorbidity clusters were identified by sex and age group (65–79 and ≥80 years).

**Results:**

322,328 patients with multimorbidity were included in the analysis (mean age, 75.4 years [Standard deviation, SD: 7.4], 57.4% women; mean of 7.9 diagnoses [SD: 3.9]). For both men and women, the first cluster in both age groups included the same two diagnoses: *Hypertensive diseases* and *Metabolic disorders*. The second cluster contained three diagnoses of the musculoskeletal system in the 65- to 79-year-old group, and five diseases coincided in the ≥80 age group: varicose veins of the lower limbs, senile cataract, dorsalgia, functional intestinal disorders and shoulder lesions. The greatest overlap (54.5%) between the three most common diagnoses was observed in women aged 65–79 years.

**Conclusion:**

This cluster analysis of elderly primary care patients with multimorbidity, revealed a single cluster of circulatory-metabolic diseases that were the most prevalent in both age groups and sex, and a cluster of second-most prevalent diagnoses that included musculoskeletal diseases. Clusters unknown to date have been identified. The clusters identified should be considered when developing clinical guidance for this population.

## Introduction

Increased life expectancy and improved health records systems have resulted in an increased population with diagnosed comorbidities. It is estimated that more than 95% of people older than 65 years in western countries will have coexisting diagnoses of two or more diseases at some point in time [1–5].

Multimorbidity (MM) measurement is a complex topic; one of the approaches that have been used to address it is to find the associations or patterns of diseases that tend to co-occur beyond the rate of chance. Systematic reviews have reported a range of statistical techniques used (prevalence figures, conditional count, odds and risk ratios, observed/expected ratio, factor analysis, cluster analysis, etc.) to identify MM patterns [1,2]. However, most of these analyses have been based on a restricted a priori list of clinical diagnoses [1,3]. Furthermore, few studies have differentiated the patterns by age and sex [1], although it is well known that differences based on these characteristics exist both in epidemiological profiles and in clinical care. A better approach to studying MM must be defined that includes a wide range of clinical diagnoses and is stratified by age and sex [3,5,6].

Cluster analysis can be used to identify patterns by establishing similarities within subgroups, with each subgroup characterized by a different profile; this is a useful method when the number and nature of the groupings is unknown a priori [7,8]. Cluster analyses have previously been used to discover MM patterns, but none considered the full range of diseases treated in Primary Care [1]. The identification of such patterns is essential to improve our knowledge about pathophysiological pathways shared by MM conditions, to guide the clinical and pharmaceutical management of these patients, and to support policy makers in the efficient allocation of resources and the design of effective health programs aimed at improving holistic, person-centred, primary health care [9,10].

Our hypothesis was that analysis of a large electronic health records (EHR) database containing all diagnoses pertaining to individual patients would identify new associations between diseases commonly present in MM patients and could improve care provided to patients who fit the profile of subgroups with these patterns. The purpose of this study was to identify clusters of diagnoses in elderly patients with MM in the primary health care system in Catalonia, by sex and age group (65–79 years and ≥80 years).

## Materials and Methods

### Design, setting and study population

**Fig 1:**
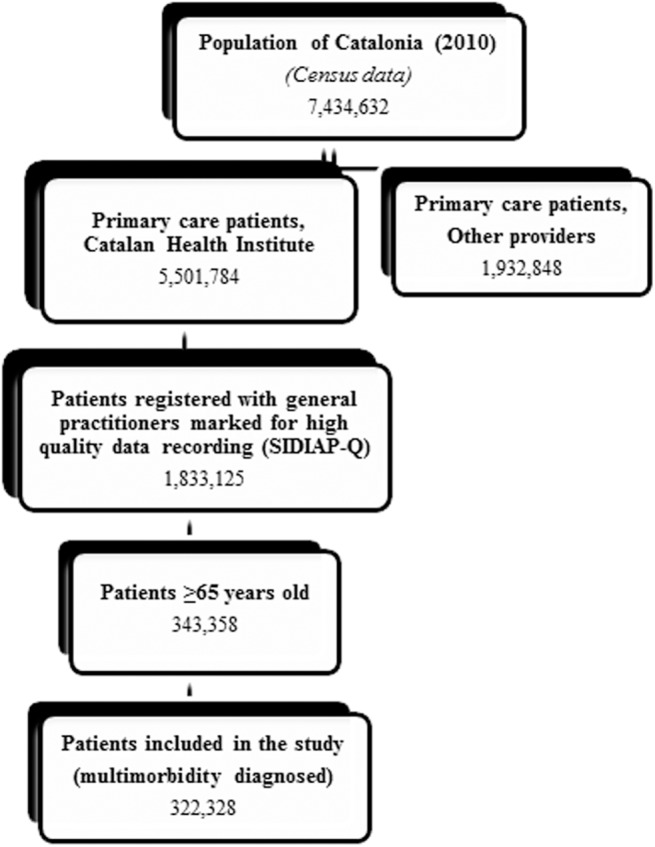
Sampling framework. Abbreviations: PHCT, primary health care teams; SIDIAP-Q, Information System for the Development of Research in Primary Care- Quality

A cross-sectional study was conducted in Catalonia (Spain), a Mediterranean region with 7,434,632 inhabitants, 81% of which live in urban municipalities (2010 census). The Spanish National Health Service (NHS) provides universal coverage, financed mainly by tax revenue. The Catalan Health Institute (CHI) manages primary health care teams (PHCTs) that serve 5,501,784 patients (274 PHCT), or 74% of the population; the remaining PHCTs are managed by other providers. The CHI’s Information System for the Development of Research in Primary Care (SIDIAP) contains the coded clinical information recorded in EHR by its 274 PHCTs since 2006. A subset of records meeting the highest quality criteria for clinical data (SIDIAP Q) includes 40% of the SIDIAP population (1,833,125 individuals), attended by the 1,365 general practitioners (GPs) whose data recording scored highest in a validated comparison process [11]. We selected individuals older than 64 years on 31 December 2010 with two or more diagnoses (Fig 1).

### Coding and selection of diseases

Diseases are coded in SIDIAP using International Classification of Diseases version 10 (ICD-10). For this study, we selected all active diagnoses recorded in EHR as of December 31, 2010, except for R codes (symptoms, signs, and abnormal clinical and laboratory findings, not elsewhere classified) and Z codes (factors influencing health status and contact with health services). Non-active diagnoses were excluded, based on the presence of an end date in the EHR. These diagnoses cover a broad list of acute diseases for which the system automatically assigns an end date (e.g., 60 days after the initial diagnosis).

To facilitate management of the diagnostic information, the diagnoses were extracted using the 263 blocks (disease categories) in the ICD-10 structure. These are homogeneous categories of very closely related specific diagnoses (for example, *Hypertensive diseases* includes Essential (primary) hypertension, Hypertensive heart disease, Hypertensive renal disease, Hypertensive heart and renal disease and Secondary hypertension). Throughout the manuscript, the ICD diagnostic blocks are italicized, followed by the most frequent specific diagnosis in parentheses where applicable. To obtain consistent and clinically interpretable patterns of association, and to avoid spurious relationships that could bias the results, we considered only diagnoses with greater than 1% prevalence in each stratum of age and sex. All patients with MM (2 or more coexisting diagnoses recorded in the EHR on 31 December 2010) were included.

### Statistical analysis

Analyses were stratified by sex and age group (65–79 and ≥80 years). In a descriptive analysis, categorical variables were expressed as frequencies (percentage) and continuous variables as mean (Standard deviation, SD) or median (interquartile range, IQR).

A cluster analysis was performed in order to identify patterns of MM. This analysis allows the assignment of diagnoses into groups or clusters, so that diagnoses in the same cluster are more similar to one another than to diagnoses from different clusters. The unit of measurement was the diagnosis (values: 1 for present, 0 for absent). In order to assess how ‘close’ the diagnoses were to each other, a quantitative measure of closeness (or similarity) for binary data, the Jaccard coefficient, was used. This coefficient considers only the diagnoses that any two patients have and ignores the diagnoses that neither of them has [7].

As we do not know a priori the number of clusters to retain from the data, we used agglomerative hierarchical methods to identify possible clustering solutions: Average linkage, Ward, flexible beta and other methods with less bias, based on nonparametric estimates, such as Single Linkage and Density Linkage. All but Ward and the flexible beta methods successively chained the observations into one cluster. Therefore, the Ward method, which minimizes the variance within clusters and produces clusters of similar sizes, was chosen as the primary method based on dendrograms analysis [7].

Data were randomly split into test and training datasets, equal in size and analysed separately. We ran the Ward method on both samples. The semi-partial R2, Calinski-Harabasz pseudo-F- and pseudo-T2-statistic criteria for different numbers of clusters were examined [7]. Clustering solutions were compared between the test and training datasets, taking into account the number of clusters, Adjusted Rand Index and clinical criteria. After checking algorithm stability, Ward method was run on the full data set, applying the same criteria to different numbers of clusters. Results were compared with flexible beta results, with beta values set at -0.25 and -0.5. The criteria for selecting the number of clusters were the highest adjusted Rand index with a high number of clusters and a high pseudo T2 statistic [12]. Clinical criteria were used to evaluate the consistency and utility of the final cluster solution, based on clusters previously described in the literature and a consensus opinion drawn from the clinical experience of the research team (4 family physicians, 1 epidemiologist in daily patient care.

After identifying the clusters of diagnoses, the patients were assigned to clusters. We considered that a patient belonged to a given cluster if he/she had one or more of the diagnoses in that cluster. Thus, a patient could belong to more than one cluster. In order to facilitate interpretation, we also calculated cluster prevalence (overall and stratified by sex and age group), further restricting the assignment of patients to those with ≥2 diagnoses. The prevalence of each specific diagnosis was calculated by stratum and cluster.

To assess the internal cluster quality, we applied multiscale bootstrap resampling to obtain an approximately unbiased (AU) probability. This probability (‘p-value’) is the proportion of bootstrapped samples that contain the cluster; larger p-values indicate more support for the cluster [13].

For the purpose of illustration, we used Venn diagrams to show the overlap between the three most prevalent clusters in each stratum.

The analyses were performed using SPSS for Windows, version 18 (SPSS Inc., Chicago, IL, USA), SAS 9.2 for Windows (SAS Institute Inc., Cary, NC, USA) and R version 3.0.0 (R Foundation for Statistical Computing, Vienna, Austria).

### Ethical considerations

The study protocol was approved by the Committee on the Ethics of Clinical Research, Institut Universitari d’Investigació en Atenció Primària Jordi Gol (IDIAP Jordi Gol) (Protocol No: P12/28). All data were anonymized and EHR confidentiality was respected at all times in accordance with national and international law.

## Results

### Population description

A total of 343,358 individuals older than 64 years (57.4% women) were selected from Primary Care records. Of these, 322,328 (93.9%) met the MM criteria and were included in the cluster analysis. Mean age of MM patients was 75.4 years (standard deviation [SD]: 7.4), with a mean of 7.9 (SD: 3.9) diagnoses per patient. The group aged ≥80 years had only marginally higher MM than the younger group (94.9% *vs* 93.4%, respectively; p<0.001). Sex-related differences were found in both age strata. In participants aged 65–79 years, women had higher MM than men (94.1% *vs* 92.7%, respectively; p<0.001), but the reverse was true among those ≥80 years old (95.3% in men *vs* 94.7% in women; p<0.001) (Table 1).

**Table 1:** Multimorbidity in patients ≥65 years old, stratified by age group and sex.

Included in the analysis N = 322,328				
	Age Group 65–79 years		Age Group ≥80 years	
	Female	Male	Female	Male
	(n = 132,553)	(n = 111,630)	(n = 63,554)	(n = 35,621)
Number of diagnoses				
2	5,582 (4.5%)	6,195 (6.0%)	2,234 (3.7%)	1,162 (3.4%)
*3*	8,150 (6.5%)	8,656 (8.4%)	3,288 (5.5%)	1,802 (5.3%)
4	10,307 (8.3%)	10,728 (10.4%)	4,398 (7.3%)	2,589 (7.6%)
≥5	100,623(80.7%)	77,933 (75.3%)	50,278 (83.5%)	28,403 (83.6%)
Median number of diagnoses (IQR)	8 (5–11)	7 (5–9)	8 (5–11)	8 (5–11)
**≥2 Diagnoses**	124,662 (94.1%)	103,512 (92.7%)	60,198 (94.7%)	33,956 (95.3%)

Abbreviations: IQR, Inter-quartile Range.

Note: P-values were significant at <0.001 (Chi-square test) in all comparisons except in number of diagnoses between females and males at age group ≥80 years.

### Identification of clusters of diagnoses

The number of clusters identified across all age and sex strata ranged from 42 to 85; however, the number of clusters with two or more diagnoses varied from 6 to 18 (Table 2). Clusters containing a single diagnosis had very low prevalence rates.

**Table 2:** Descriptive analysis of clusters and their structure in multimorbidity patients ≥ 65 years old, stratified by age group and sex.

Age group	Sex	Number of diagnoses	Number of clusters	Number of clusters with ≥2 diagnoses	Median of diganoses per clusters (IQR)*
65–79	Female	94	42	18	2 (2–5)
	Male	88	67	11	2 (2–3)
≥80	Female	99	85	6	2 (2–4)
	Male	99	58	18	3 (2–4)

Abbreviations: IQR, Inter-quartile Range

*Median of clusters with ≥2 diagnoses.

The vast majority of patients (63.2%-81.4%) had at least two diagnoses included in the most prevalent cluster (28.1% to 47.3% for the second most prevalent cluster). There was no consistency, however, in the specific composition of the most prevalent clusters across strata (Tables 3–6). In order to simplify the presentation of the results, we describe only the four most prevalent clusters. The description of all the clusters, using the Ward algorithm, is shown in S1–S4 Tables.

**Table 3:** Four most prevalent clusters of diagnoses: Prevalence and composition of clusters in women aged 65–79 years (n = 124,662).

Cluster rank	Number of patients	Diagnosis prevalence in stratum (%)		Diagnoses	Prevalence (%)	
		≥ 1 diagnosis	≥ 2 diagnosis		In stratum	In cluster
**1**	113.667	91.2	67.8	Hypertensive diseases	64.0	70.2
** **				Metabolic disorders	57.7	63.2
** **				Arthrosis	41.3	45.3
** **				Obesity and other hyperalimentation	26.5	29.1
** **				Diabetes mellitus	21.9	24.0
**2**	96.131	77.1	45.8	Other dorsopathies	32.2	41.7
** **				Disorders of bone density and structure	27.5	35.7
** **				Other soft tissue disorders	26.8	34.8
** **				Diseases of veins, lymphatic vessels and lymph nodes, not elsewhere classified	26.7	34.7
** **				Neurotic, stress-related and somatoform disorders	21.4	27.8
** **				Other joint disorders	19.7	25.5
**3**	92.539	74.2	42.2	Other forms of heart disease	16.9	22.8
** **				Mood [affective] disorders	16.3	22.0
** **				Disorders of thyroid gland	15.6	21.0
** **				Other diseases of intestines	14.5	19.6
** **				Disorders of lens	14.5	19.5
** **				Diseases of oesophagus, stomach and duodenum	14.0	18.8
** **				Chronic lower respiratory diseases	12.5	16.8
** **				Noninflammatory disorders of female genital tract	12.5	16.8
** **				Benign neoplasms	11.5	15.5
** **				Hernia	10.7	14.4
** **				Glaucoma	8.4	11.3
**4**	68.431	54.9	22.5	Diseases of oral cavity, salivary glands and jaws	12.0	21.9
** **				Other disorders of ear	11.0	20.0
** **				Acute upper respiratory infections	10.6	19.4
** **				Dermatitis and eczema	9.9	18.0
** **				Behavioural syndromes associated with physiological disturbances and physical factors	8.4	15.3
** **				Episodic and paroxysmal disorders	8.4	15.3
** **				Other diseases of urinary system	8.1	14.8
** **				Other diseases of upper respiratory tract	7.4	13.5
** **				Nerve, nerve root and plexus disorders	6.6	12.0
** **				Mycoses	6.0	10.9

AU p-value: cluster 1: 0.99 (0.83–1); cluster 2: 1.00; cluster 3: 0.96 (0.95–0.98); cluster 4: 0.91 (0.87–0.94).

**Table 4:** Four most prevalent clusters of diagnoses: Prevalence and composition of clusters in men aged 65–79 years (n = 103,512).

Cluster rank	Number of patients	Diagnosis prevalence in stratum (%)		Diagnoses	Prevalence (%)	
		≥ 1 diagnosis	≥ 2 diagnosis		In stratum	In cluster
**1**	92.419	89.3	63.2	Hypertensive diseases	60.6	67.9
** **				Metabolic disorders	52.6	58.9
** **				Diseases of male genital organs	36.1	40.4
** **				Diabetes mellitus	27.8	31.1
** **				Obesity and other hyperalimentation	16.6	18.6
**2**	63.964	61.8	28.1	Other dorsopathies	25.7	41.6
** **				Arthrosis	22.7	36.7
** **				Other soft tissue disorders	18.1	29.3
** **				Diseases of oral cavity, salivary glands and jaws	13.0	21.0
** **				Other joint disorders	11.9	19.3
** **				Other disorders of ear	11.6	18.8
**3**	35.334	34.1	5.6	Chronic lower respiratory diseases	21.5	62.9
** **				Mental and behavioural disorders due to psychoactive substance use	18.2	53.4
**4**	31.144	30.1	4.6	Other forms of heart disease	21.1	70.1
** **				Ischaemic heart diseases	13.6	45.2

AU p-value: cluster 1: 0.76 (0.71–0.81); cluster 2: 0.77 (0.72–0.82); cluster 3: 1.00; cluster 4: 1.00.

**Table 5:** Four most prevalent clusters of diagnoses: Prevalence and composition of clusters in women aged≥80 years (60,198).

Cluster rank	Number of patients	Diagnosis prevalence in stratum (%)		Diagnoses	Prevalence (%)	
		≥ 1 diagnosis	≥ 2 diagnosis		In stratum	In cluster
**1**	56.509	93.9	71.2	Hypertensive diseases	77.4	82.4
** **				Metabolic disorders	50.5	53.7
** **				Arthrosis	46.9	50.0
** **				Other forms of heart disease	33.3	35.5
**2**	46.21	76.8	47.3	Diseases of veins, lymphatic vessels and lymph nodes, not elsewhere classified	25.7	33.5
** **				Disorders of lens	25.0	32.6
** **				Disorders of bone density and structure	23.6	30.8
** **				Other dorsopathies	22.5	29.2
** **				Other diseases of intestines	19.7	25.7
** **				Neurotic, stress-related and somatoform disorders	17.0	22.2
** **				Other soft tissue disorders	16.0	20.8
** **				Other joint disorders	14.1	18.3
**3**	22.592	37.5	6.8	Diabetes mellitus	24.3	64.7
** **				Obesity and other hyperalimentation	20.1	53.5
**4**	13.406	22.3	2.5	Obestity and other hyperalimentation	12.7	36.9
** **				Diseases of oesophagus, stomach and duodenum	12.6	56.4
** **				Hernia	12.2	54.7

AU p-value: cluster 1: 1.00; cluster 2: 0.78 (0.73–0.83); cluster 3: 1.00; cluster 4: 0.61 (0.54–0.67).

**Table 6:** Four most prevalent clusters of diagnoses: Prevalence and composition of clusters in men aged ≥80 years (n = 33,956).

Cluster rank	Number of patients	Diagnosis prevalence in stratum (%)		Diagnoses	Prevalence (%)	
		≥ 1 diagnosis	≥ 2 diagnosis		In stratum	In cluster
**1**	32.608	96.0	81.4	Hypertensive diseases	67.2	70.0
** **				Diseases of male genital organs	44.0	45.8
** **				Metabolic disorders	43.0	44.8
** **				Other forms of heart disease	37.9	39.5
** **				Arthrosis	30.9	32.2
** **				Chronic lower respiratory diseases	27.9	29.0
** **				Diabetes mellitus	27.0	28.1
**2**	24.012	70.7	38.5	Disorders of lens	23.2	32.8
** **				Other dorsopathies	20.9	29.6
** **				Other diseases of intestines	18.4	26.0
** **				Hernia	16.9	23.8
** **				Diseases of oesophagus, stomach and duodenum	15.4	21.8
** **				Diseases of veins, lymphatic vessels and lymph nodes, not elsewhere classified	14.2	20.1
** **				Other disorders of ear	13.9	19.6
** **				Other soft tissue disorders	12.3	17.3
**3**	13.538	39.9	10.9	Ischaemic heart diseases	18.9	47.5
** **				Renal failure	16.0	40.2
** **				Diseases of arteries, arterioles and capillaries	10.6	26.5
** **				Aplastic and other anaemias	7.5	18.7
**4**	11.668	34.4	5.8	Obestity and other hyperalimentation	12.7	36.9
** **				Glaucoma	10.2	29.8
** **				Inflammatory polyarthropaties	9.8	28.3
** **				Diseases of external ear	8.0	23.4

AU p-value: cluster 1: 0.80 (0.76–0.85); cluster 2: 0.81 (0.76–0.86); cluster 3: 0.81 (0.76–0.81); cluster 4: 0.74 (0.64–0.84).

Several patterns were identified. The most prevalent cluster in all four strata included *Hypertensive diseases* and *Metabolic disorders* and tended to include other age-related and/or cardiovascular diagnoses. In the younger group (both sexes), *Diabetes mellitus* and *Obesity and other hyperalimentation* were also included. In the older group (both sexes), the most prevalent cluster included *Other forms of heart disease* (atrial fibrillation) as a third diagnosis. *Arthrosis* was part of this cluster in three strata, but was replaced by *Diseases of male genital organs in males* (hyperplasia of prostate) in men aged 65–79 years.

The second most prevalent cluster included diagnoses related to the musculoskeletal system: *Other dorsopathies* (dorsalgia), *Other soft tissue disorders* (shoulder injuries), and *Other joint disorders*. In women of both age groups, the cluster included two aging-related disorders: *Disorders of bone density and structure* (osteoporosis) and *Disorders of lens* (cataracts).

The cluster of third-most prevalence covers a range of diagnoses (Tables 3 and Table 6). In 65- to 79-year-olds, however, *Chronic lower respiratory diseases* and *Mental and behavioural disorders due to psychoactive substance use* (Tobacco) had a higher prevalence in men (Table 4). Two diagnoses were prevalent in women ≥80 years old: *Diabetes mellitus* and *Obesity and other hyperalimentation*.

**Fig 2:**
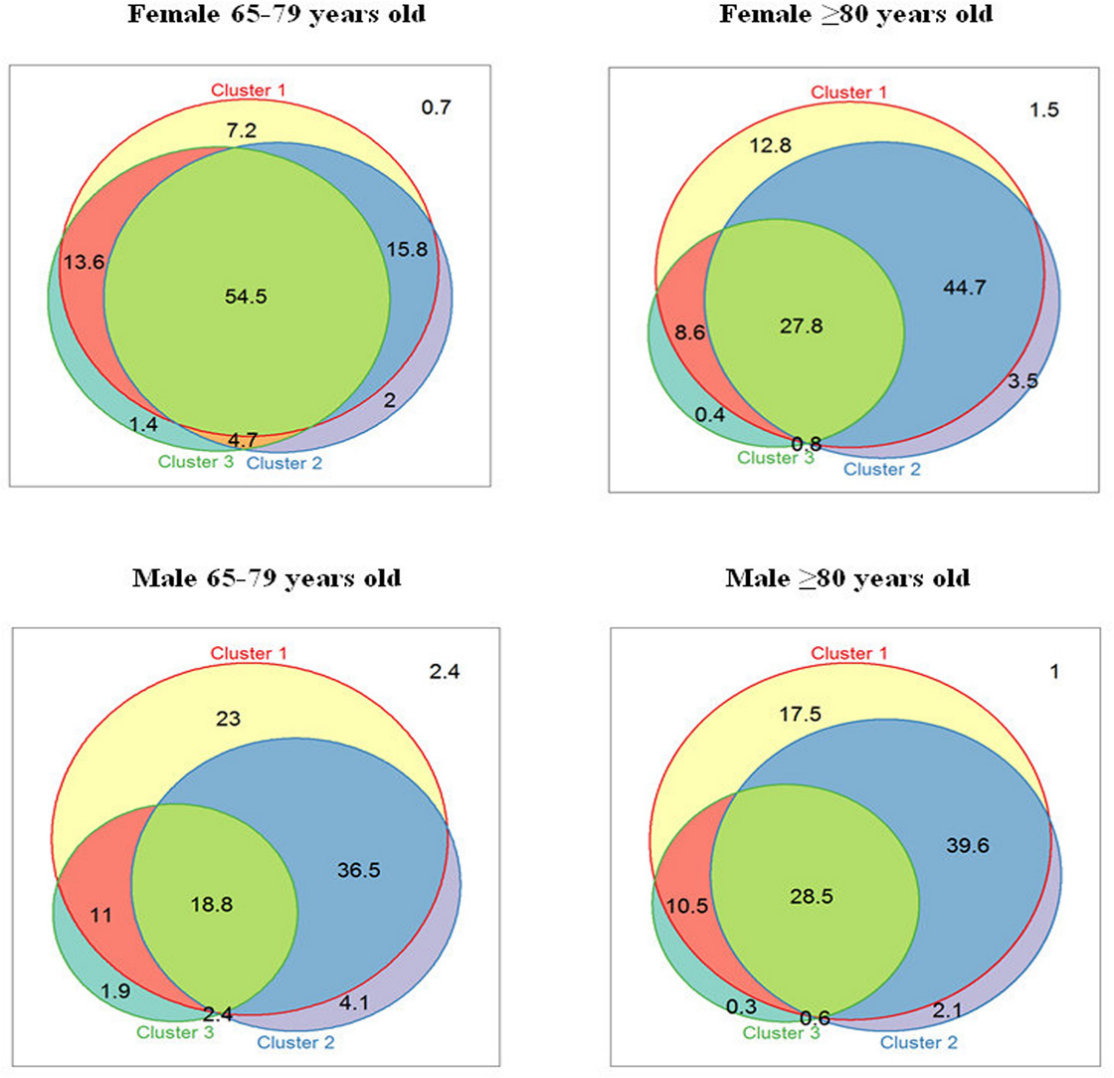
Percentage of individuals (%) with overlapping of the three most prevalent clusters by sex and age group.

We present Venn diagrams for the overlap of the three most prevalent clusters across all patients (Fig 2). Less than 2.5% of all patients in any group were free of all diagnoses in the three most prevalent clusters. The group with the largest overlap was women aged 65 to 79 years: 54.5% presented with diagnoses in all three clusters. The least overlap (18.8%) was observed in men of that age group. In general, women had a higher frequency of overlap between clusters (≥81.8%) than men (≥68.6), p<0.001.

## Discussion

We observed a very high prevalence of multimorbidity among elderly individuals in primary care. Cluster analysis, including all conditions above a minimum prevalence threshold, showed some well-known multimorbidity patterns and revealed others that have not been described previously. We detected substantial heterogeneity in the composition of multimorbidity clusters across age/sex strata, but several distribution patterns emerged: a) a circulatory-metabolic cluster was the most prevalent in all age groups, followed by a cluster that included mostly musculoskeletal diagnoses; b) aging-related diagnoses were consistently included in each of the four most frequent clusters in all age/sex strata; and c) sex related differences in the distribution of multimorbidity were observed for the younger stratum patients but not for the older groups.

The patterns of associations between diagnoses are consistent with previous studies focussing on different populations and using other methodologies, such as the association between cardiovascular, endocrine and metabolic diseases [8, 14–17]. In our study, arthrosis tended to be clustered with diabetes and hypertension. Similarly, arthropathies (arthritis) have been identified along with diabetes and hypertension in participants older than 50 years [18]. The association of prostatic hyperplasia with endocrinal and cardiovascular diseases has been described in the same population using different statistical methods [19]. In our study, the musculoskeletal diagnoses included anxiety, limb varicose veins and osteoporosis in women. A very similar pattern was described previously by Prados et al [3,20]. An association between musculoskeletal disorders and gastro-esophageal reflux disease (GERD) was described by Cornell et al [8]. The relationships between these pathologies may be explained by the observed association between chronic pain and mental disorders [21,22], but also, as previously suggested, by gender stereotypes in the diagnostic process [23]. Our results suggest several possible pathophysiologic explanations for some of the observed associations that have not been previously reported, likely due to the limited number of clinical diagnoses assessed in earlier studies. For example, the musculoskeletal cluster in males aged 65 to 79 years also includes hearing loss; diseases of the oral cavity might be explained by bone degeneration and arthrosis of the small joints. The musculoskeletal/digestive cluster in older men included a range of digestive pathologies (haemorrhoids, diseases of the oesophagus and inguinal hernia) that have a better-known association with GERD than with musculoskeletal problems. Although this could be explained by aging-related changes in connective tissue that may be a risk factor for both skeletal and extraskeletal disorders (varicose veins, aneurysms, hernias, myopia, etc.) [24], other clusters are difficult to interpret from a pathophysiological point of view.

We note the higher overlap of clusters observed for women, particularly those aged 65–79 years. Previous studies have reported higher MM rates in women [6], but this was not the case for our older age group, in which men had marginally but significantly higher MM levels.

Elderly patients had a median of 7 to 8 diseases, indicating a need for appropriate methods of grouping diseases beyond combinations of 2 or 3 highly prevalent diseases in order to study the complexity of multimorbidity in this population. Although various approaches (cluster analysis, factor analysis, latent class analysis, etc.) have been applied to date, there is no agreement about which is the most accurate [1, 25]. The present data provide information about the application of the cluster method in-depth to a large sample of diseases in order to advance the discussion about which of these methods might be the most recommended for the study of multimorbidity.

The clusters here identified can be used to prioritize interventions addressing some of the most common problems encountered in primary care, such as disorganisation and fragmentation of care, the improvement of current specific guidelines, challenges in delivering patient- centred care and barriers to shared decision making [26].

### Strengths and limitations

The major strength of this study is the analysis of a large, high-quality database of primary-care records that have been shown to be representative of a much larger population. Other studies have shown that more accurate conclusions can be drawn from EHR data than from survey-based datasets [27–29]. Analysing almost all potential diagnoses could have added a complexity that may hinder interpretation of findings and comparison with other studies.

This wide range of diagnoses stratified by sex and age allowed us to find associations that have been little studied or have not been suggested before. Unlike previous studies, we did not explore only the associations between diagnoses but also the distribution of the resulting clusters in the studied population. This provided empirical evidence of clinical relevance and offers an approach to MM analysis that is patient-centred, rather than disease-centred.

A number of limitations need to be taken into account as well. Cluster analysis is exploratory in nature, and different clustering algorithms may produce different results [30]. The final clustering solution presented here was obtained through a systematic and rigorous process, including comparing the results from a randomly split dataset, testing different clustering algorithms, using different objective numeric criteria to decide the number of clusters, as well as subjective clinical criteria applied by a panel of experts in order to assess whether the groupings were clinically interpretable. An important limitation is our use of agglomerative hierarchical clustering, which forces every unit (i.e., diagnosis) into a single cluster. Hierarchical algorithms are considered more appropriate for classification problems that share common underlying factors, and may be a useful starting point when the number and structure of the clusters is unknown [7]. Another limitation is our use of ICD-10 3-character codes as the unit of analysis, rather than the more specific individual diagnosis.

### Implications for clinical practice and policy

Longitudinal and genetic studies are needed to confirm or refine the observed patterns, which would give clinicians and policy makers now have access to information on how diseases are clustered in the older adult population. This is important for developing disease-specific Clinical Practice Guidelines that appropriately reflect the co-occurrence of conditions in this population and can anticipate tailored approaches based on the comorbidity profile of the individual patient. In day-to-day clinical practice, this information is useful to increase clinical suspicion and case-finding of conditions within the same cluster when considering the differential diagnosis of new health concerns.

### Future research

To further assess the stability of these clusters over time and confirm that the observed results are not simply due to chance, longitudinal studies are needed and the new clinical hypotheses should be tested. Longitudinal studies would show when second- and third- level diseases are added to first-level diagnoses during the individual's lifetime. They would also allow exploration of factors that produce or lead to comorbidity; these data could be used to design individualized preventive strategies. Analysis is needed of potential confounding factors such as greater disease severity, socioeconomic status, place of residence, comorbid conditions, or functional limitations [10]. Clinical studies could assess clusters that are biologically plausible or have an unknown clinical relationship, but are potentially important for clinical practice. Clinical trials are needed to determine which therapeutic approaches best address the most prevalent clusters and to develop prevention strategies based on these clusters. Additional research priorities should be to explore the impact of these disease clusters on patients’ quality of life, activities of daily living and prognosis [31]. These clusters have to be tested with networks methods to identify genetic abnormalities or the interplay of multiple molecular processes that may be involved with multimorbidity [32]. Future replication in other databases from other countries is also needed in order to explore the external validity of the present findings and to assess whether the multimorbidity patterns obtained could be generalized to multimorbidity patients in a broader context.

## Conclusions

We identified several clusters of diagnoses that are most prevalent by age group and sex in older adults. Some of these clusters were not previously observed but show a high degree of consistency across all strata. This study included a broad range of diagnoses, and corroborated some clusters of diseases that do not co-occur by chance. In all strata, hypertensive diseases and metabolic disorders consistently made up the most prevalent cluster, followed by the musculoskeletal diseases cluster. The results of this study offer the opportunity to shape future research on combined preventive measures for the different conditions within a given cluster and to inform clinical practice guidelines as well as diagnostic procedures and algorithms in the primary care setting.

In summary, the identification of MM patterns facilitates the holistic approach to health care, focusing not only on a specific disease, but on the whole person and health promotion. The results of our study add knowledge to encourage this paradigm shift in health care.

## Supporting Information

S1 Table Prevalence and composition of diagnostic clusters in women aged 65–79 years. (XLSX)

S2 Table Prevalence and composition of diagnostic clusters in men aged 65–79 years. (XLSX)

S3 Table Prevalence and composition of diagnostic clusters in women aged ≥80 years. (XLSX)

S4 Table Prevalence and composition of diagnostic clusters in men aged ≥80 years. (XLSX)
